# The Spatiotemporal Pattern of Glis3 Expression Indicates a Regulatory Function in Bipotent and Endocrine Progenitors during Early Pancreatic Development and in Beta, PP and Ductal Cells

**DOI:** 10.1371/journal.pone.0157138

**Published:** 2016-06-07

**Authors:** Hong Soon Kang, Yukimasa Takeda, Kilsoo Jeon, Anton M. Jetten

**Affiliations:** Cell Biology Group, Immunity, Inflammation, and Disease Laboratory, Division of Intramural Research, National Institute of Environmental Health Sciences, National Institutes of Health, Research Triangle Park, 27709, NC, United States of America; INSERM UMRS 1138, FRANCE

## Abstract

The transcription factor Glis-similar 3 (Glis3) has been implicated in the development of neonatal, type 1 and type 2 diabetes. In this study, we examined the spatiotemporal expression of Glis3 protein during embryonic and neonatal pancreas development as well as its function in PP cells. To obtain greater insights into the functions of Glis3 in pancreas development, we examined the spatiotemporal expression of Glis3 protein in a knockin mouse strain expressing a Glis3-EGFP fusion protein. Immunohistochemistry showed that Glis3-EGFP was not detectable during early pancreatic development (E11.5 and E12.5) and at E13.5 and 15.5 was not expressed in Ptf1a^+^ cells in the tip domains indicating that Glis3 is not expressed in multipotent pancreatic progenitors. Glis3 was first detectable at E13.5 in the nucleus of bipotent progenitors in the trunk domains, where it co-localized with Sox9, Hnf6, and Pdx1. It remained expressed in preductal and Ngn3^+^ endocrine progenitors and at later stages becomes restricted to the nucleus of pancreatic beta and PP cells as well as ductal cells. Glis3-deficiency greatly reduced, whereas exogenous Glis3, induced Ppy expression, as reported for insulin. Collectively, our study demonstrates that Glis3 protein exhibits a temporal and cell type-specific pattern of expression during embryonic and neonatal pancreas development that is consistent with a regulatory role for Glis3 in promoting endocrine progenitor generation, regulating insulin and Ppy expression in beta and PP cells, respectively, and duct morphogenesis.

## Introduction

Progressive loss and/or dysfunction of pancreatic beta cells underlie all types of diabetes and include abnormalities in insulin regulation and changes in the developmental programming of beta cells. Both genetic and environmental factors have been implicated in the development of diabetes. The control of pancreas development and insulin expression is complex and regulated by many transcription factors. Recently, Gli-similar 3 (Glis3) was identified as a novel critical regulator of pancreatic beta cell generation and insulin expression [1–7]. Glis3 belongs with Glis1 and -2 to a subfamily of Krüppel-like zinc finger transcription factors that share a conserved zinc finger domain (ZFD) consisting of five Cys_2_-His_2_ zinc finger motifs [2, 7–9]. The ZFD plays a critical role in the recognition of specific DNA elements, referred to as Glis-binding sites or GlisBS, in the regulatory region of target genes. Genetic aberrations in human *GLIS3* are associated with a syndrome that is characterized by neonatal diabetes and hypothyroidism (NDH) and may include polycystic kidney disease, glaucoma, and mild mental retardation depending on the nature of the mutation [10, 11]. In addition, genome-wide association studies (GWAS) reported an association between single nucleotide polymorphisms at the *GLIS3* gene locus with an increased risk for developing type 1 and 2 diabetes [12–16]. As in humans, mice defective in Glis3 function develop neonatal diabetes, hypothyroidism, and polycystic kidney disease, while heterozygous Glis3 knockout mice are more susceptible to diet-induced diabetes [1, 3–5, 17, 18].

Pancreas development is a multistep process that is defined by three major periods (primary and secondary transition, and postnatal period) starting with the formation of the dorsal and ventral pancreatic buds followed by branching and cell specification, finally resulting in the development of pancreatic acini, ducts, and islets [19–24]. Cell lineage determination during pancreas development involves the regulation by many transcription factors [19, 20, 22, 23, 25–30], including Glis3 [1–6, 10, 31, 32]. However, very little is still known about the spatiotemporal expression of Glis3 protein during embryonic and postnatal pancreatic development.

To obtain greater insights into the critical role that Glis3 plays in the pancreas and in neonatal, type 1 and type 2 diabetes, it is vital to understand its cell type-specific expression and subcellular localization. To study this, we generated a knockin mouse expressing a Glis3-EGFP fusion protein that allowed us to monitor Glis3 expression in specific cell types during embryonic pancreas development and postnatally. Our study demonstrated for the first time that during embryonic development endogenous Glis3 protein is not detectable in multipotent pancreatic progenitors, but is first observed in the nucleus of bipotent progenitors. Glis3 remains expressed in Ngn3^+^ endocrine progenitors and preductal cells, and at later stages becomes restricted to beta cells, pancreatic polypeptide (PP) cells, and cells lining the pancreatic ducts. Our data further show that, in addition to the greatly reduced insulin expression and the formation of dilated pancreatic ducts, the expression of pancreatic polypeptide (Ppy) is greatly impaired in Glis3-deficient mice. Together, our study demonstrates that Glis3 protein is expressed in a spatiotemporal manner during embryonic development and has a critical regulatory function in pancreatic beta cells, ducts and Ppy cells. These observations will help us to better understand the functions of Glis3 in the pancreas as well as in diabetes.

## Materials and Methods

### Generation of Glis3-EGFP Mice

**Fig 1:**
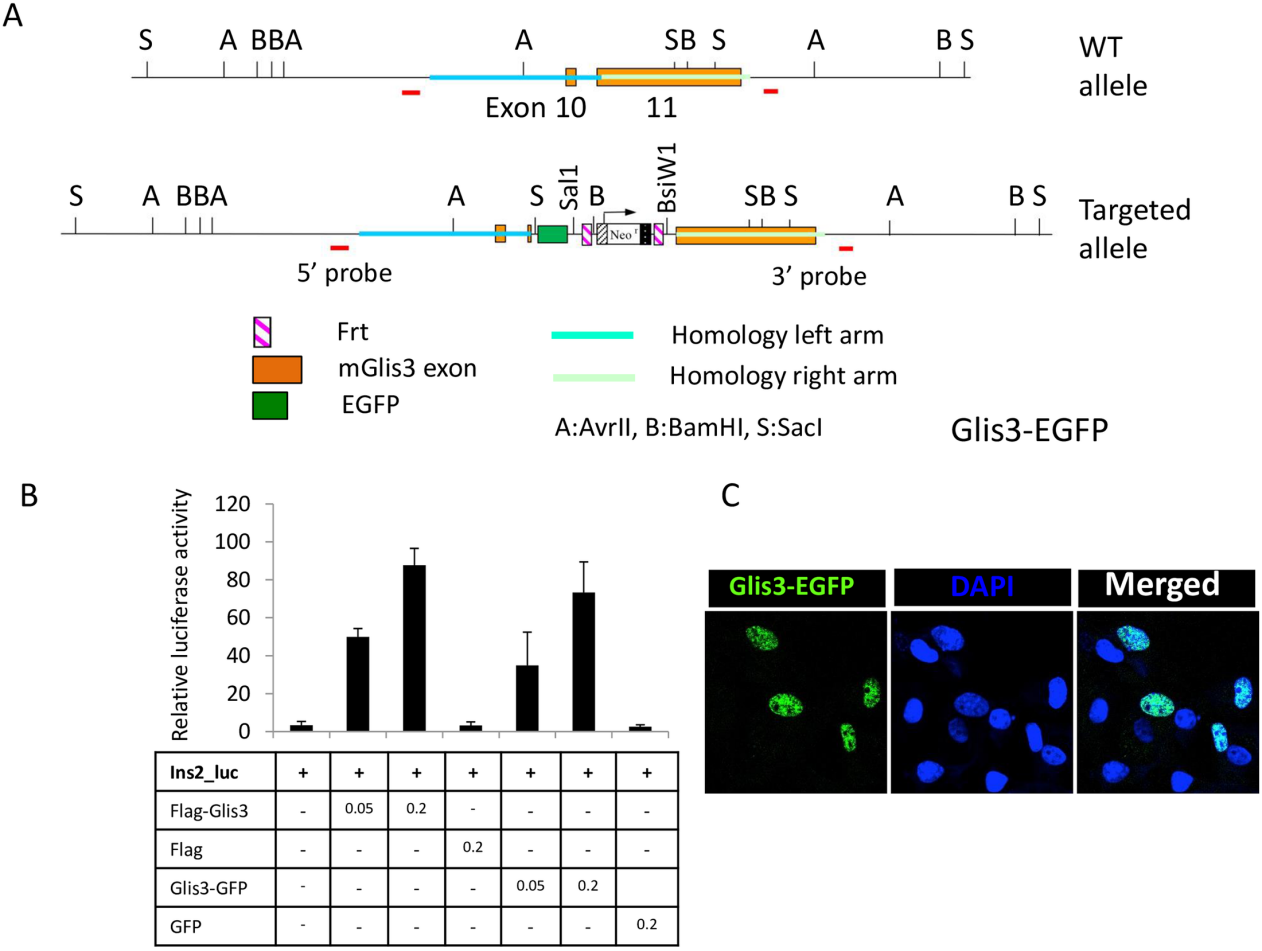
Generation of Glis3-EGFP mice. (A) Schematic view of the mouse WT Glis3 allele and the targeted allele. EGFP was inserted in frame into exon 11 just before the TGA stop codon to generate a Glis3-EGFP fusion protein. To confirm the targeting in ES clones, genomic Southern was performed with the 5’ and 3’ probes as indicated in the map. (B) Comparison of the transcriptional activity of Glis3-EGFP with that of Flag-Glis3. A luciferase reporter driven by the *Ins2* promoter (Ins2-Luc) was used to examine the transcription activity in HEK293 cells. Data shown are representative of 3 independent experiments. Each experiment was carried out in triplicate. Data present mean ± SD. There was no statistical difference in reporter activation by Flag-Glis3 and Glis3-GFP. (C) The subcellular localization of Glis3-EGFP in COS-1 cells was analyzed by confocal microscopy. DOI 10.6084/m9.figshare.3189148.

To generate Glis3^GFP/GFP^ mice, the EGFP coding region was inserted into exon 10 of *Glis3*, right before the TGA stop codon, thereby generating a Glis3-EGFP fusion protein (Fig 1A). Glis3-deficient *Glis3KO2* mice were generated by inserting *mCherry* together with a triple stop codon into exon 3 of the *Glis3* gene to ensure translational termination of Glis3 right after amino acid 158 (Kang, H.S. and Jetten, A.M., unpublished). Mice were euthanized using CO2. All animal studies followed guidelines outlined by the NIH Guide for the Care and Use of Laboratory Animals and protocols were approved by the Institutional Animal Care and Use Committee at the NIEHS.

### Cell Culture and Reporter Assays

To analyze the transcriptional activity of Glis3-EGFP, full-length of Glis3 was cloned into *Eco*RI and *Bam*HI sites of pEGFP-N1 vector (Clontech Laboratories, Mountain View, CA). Glis3-EGFP reporter assay was performed using the mIns2(-696)-Luc reporter in HEK293 cells as previously described [1]. To examine subcellular localization of Glis3-EGFP, COS-1 cells were transiently transfected with pEGFP-N1-Glis3 for 48 hrs. Then cells were fixed in 4% paraformaldehyde. Nuclei were stained with 4′,6-diamidino-2-phenylindole (DAPI). Fluorescence was observed in a Zeiss_LSM510 confocal microscope. αTC1-9 cells were obtained from American Type Culture Collection (Manassas, VA) and human pancreatic ductal epithelial cells, HPDE6-E6E76c7 (HPDE) from Dr. M-S. Tsao (Ontario Cancer Institute, Toronto). pLVX-mCherry-Glis3, -Pdx1, -MafA, -NeuroD1 lentivirus was produced as described previously [33].

### Northern Blot Analysis

Northern blot analysis was performed as described previously [34].

### Immunohistochemistry

Pancreas tissues were fixed overnight in 4% paraformaldehyde, incubated in 30% sucrose, and then embedded in O.T.C. medium (Tissue-Tek, Hatfield, PA). Frozen sections (10 microns) were prepared using a Leica cryostat (Leica) and then incubated with primary antibodies against insulin, E-cadherin (Sigma, St. Louis, MO), glucagon, somatostatin, pancreatic polypeptide (Dako, Carpinteria, CA), Glut2 (EMD Millipore, WA), Pdx1 (Abcam, Cambridge, MA), Isl-1, Sox9, Hnf6, ghrelin (Santa Cruz, CA), Ptf1a (Beta Cell Biology Consortium), Nkx2.2, Nkx6.1, Ngn3 (Developmental Studies Hybridoma Bank), Amylase (Cell Signaling Technology, Danvers, MA), and GFP (rabbit GFP antibody, Life Technologies; chicken GFP antibody, Abcam), with the FITC-conjugated lectin Dolichos biflorus agglutinin (DBA), a ductal cell marker or DAPI. After washing, the sections were subsequently incubated with anti-mouse, anti-chicken, anti-rabbit or anti-rat Alexa Fluor-488, Fluor-594, or Fluor-647 conjugated secondary antibodies (Life Technologies, Grand Island, NY). Fluorescence was observed in a Zeiss LSM780 or Zeiss LSM510 confocal microscope. Pancreata from at least 3 mice and at least 5 different sections from each pancreas were analyzed.

### Quantitative RT-PCR (QRT-PCR) Analysis

Total RNA was isolated from cell lines or mouse pancreas using an RNeasy mini kit and reverse-transcribed using a High-Capacity cDNA Archive Kit (Applied Biosystems, Foster City, CA). QRT-PCR analysis was performed using SYBR Green I or TaqMan Universal PCR Master Mix (Applied Biosystems) as described previously [34]. The data were normalized against the amount of 18S rRNA. Primers are listed in S1 Table.

### Statistics

The data generated in this study were analyzed by One-way ANOVA.

## Results

### Glis3 Is Expressed in Pancreatic Beta, PP and Ductal Cells

Although previous studies reported that Glis3 mRNA is expressed in pancreatic beta cells and in embryonic pancreas [1, 10, 35], due to the lack of a suitable Glis3 antibody very little is known about the spatiotemporal pattern of expression of Glis3 protein *in vivo*. To monitor the expression and subcellular localization of Glis3 during pancreas development, we generated an EGFP knockin mouse, Glis3^GFP/GFP^, expressing a Glis3-EGFP fusion protein (Fig 1A). Reporter assays and immunocytochemistry demonstrated that Glis3-EGFP was as transcriptionally active as Glis3 and localized to the nucleus (Fig 1B and 1C), while Northern blot analysis confirmed the expected size (9 kb) of the Glis3-EGFP transcript (S1 Fig). Up to the age of 15 months, Glis3^GFP/GFP^ mice appeared normal and did not exhibit any of the phenotypes associated with the Glis3 knockout mice reported previously [1] or any other abnormality. These results indicated that Glis3-EGFP fusion protein appeared to function normally in Glis3^GFP/GFP^ mice.

**Fig 2:**
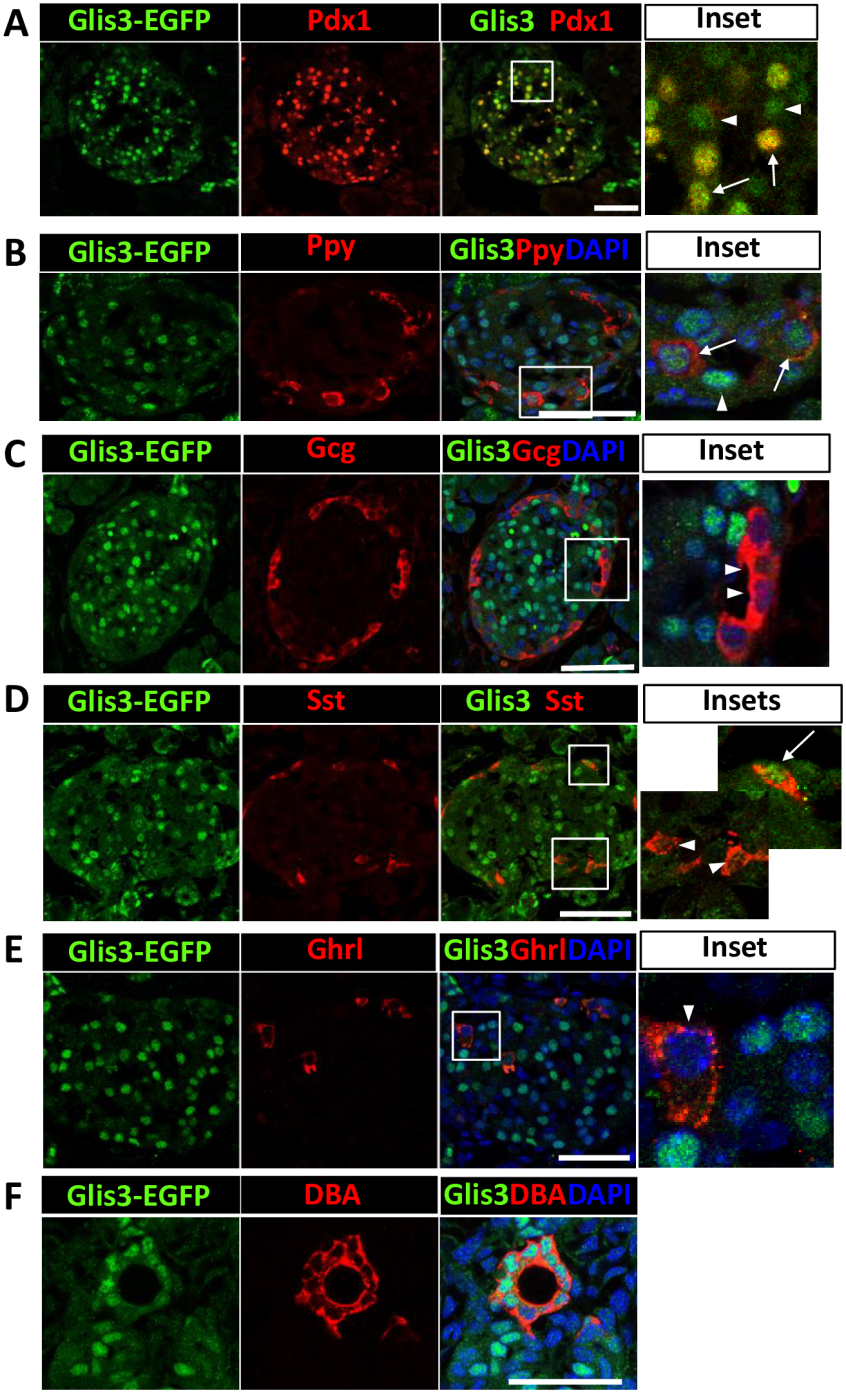
Glis3 protein is selectively expressed in mouse pancreatic islets and ducts. Pancreata from P7 Glis3^GFP/GFP^ mice were examined by immunohistochemistry with antibodies against GFP (A-E), Pdx1 (A), Ppy (B), Gcg (C), Sst (D), Ghrl (E) or stained with DBA or DAPI (F). Boxed areas were enlarged and shown in the panels on the right. Arrows indicate Glis3^+^Pdx1^+^ (A), Glis3^+^Ppy^+^ (B) or Glis3^+^Sst^+^ (D) cells; arrow heads indicate either Glis3 single positive cells (A, B) or Glis3^-^Gcg^+^ (C), Glis3^-^Sst^+^ (D) or Glis3^-^Ghrl^+^ (E) cells. Eighty four percent of Glis3^+^ cells were Pdx1^+^. More than 90% of the Ppy^+^ cells were Glis3^+,^ and more than 94% of the DBA^+^ cells were Glis3^+^. Bars indicate 50 μm. DOI 10.6084/m9.figshare.3189163.

Analysis of Glis3-EGFP protein expression in postnatal day 7 (P7) pancreas with an anti-GFP antibody showed that Glis3 protein was detected in the nucleus of the majority of Ecad^+^ islets (S2 Fig). Co-staining with an anti-Pdx1 or anti-insulin antibody showed that most Glis3^+^ cells were Pdx1^+^ (84 ± 3.48%)(Fig 2A), while 94 ± 1.6% of Glis3^+^ cells were insulin^+^ (S3 Fig) consistent with the concept that Glis3 is expressed in pancreatic beta cells. Similar results were obtained with the pancreas of P1 and P14 Glis3^GFP/GFP^ mice (data not shown). A small number of Glis3^+^ cells were not Pdx1^+^ or insulin^+^ (arrows in Fig 2A and S3 Fig) suggesting that Glis3 might be expressed in cells other than beta cells. Indeed, immunohistochemistry demonstrated that cells expressing Ppy showed nuclear expression of Glis3 (Fig 2B), but Glis3 was not detectable in Gcg^+^ alpha cells or Ghrl^+^ cells (Fig 2C and 2E) nor in acini (S4 Fig). A few Sst^+^ cells (< 10%) were found to be Glis3-EGFP^+^ (Fig 2D). In addition, 94 ± 5.1% of the DBA^+^ pancreatic ductal cells stained positively for Glis3-EGFP (Fig 2F). Together, our results demonstrate that in neonatal pancreas Glis3 is selectively expressed in the nucleus of pancreatic beta and PP cells, as well as ductal cells.

### Glis3 Is Expressed in Bipotent Progenitors and Endocrine Progenitors during Pancreatic Development

**Fig 3:**
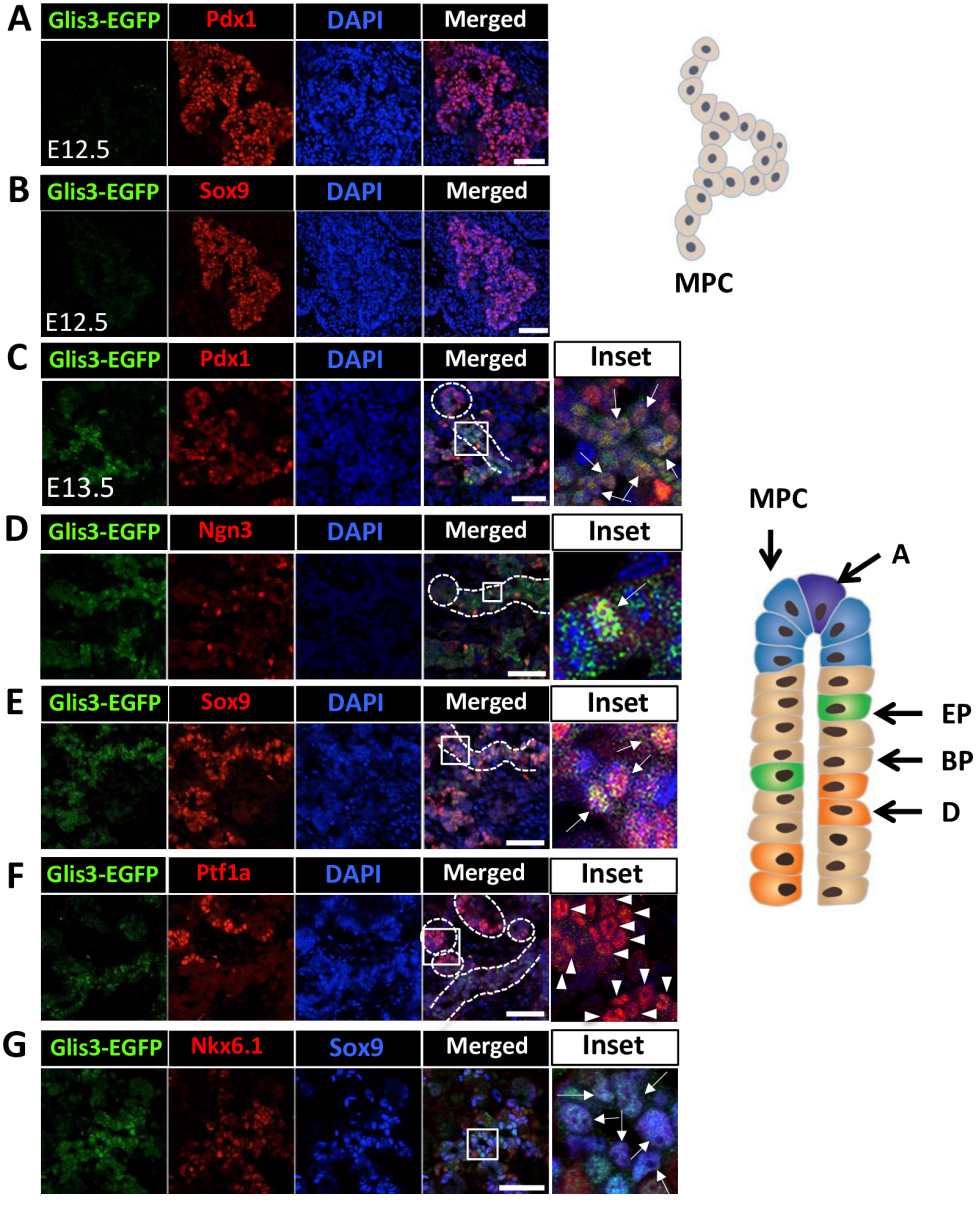
Glis3 protein was not detectable in E12.5 pancreata, but was selectively expressed at E13.5 of pancreas development. E12.5 pancreata from Glis3^GFP/GFP^ embryo were double-stained with antibodies against GFP (A-B) and Pdx1 (A) or Sox9 (B). E13.5 Glis3^GFP/GFP^ pancreata were double-stained with antibodies against GFP (C-G) and Pdx1 (C), Ngn3 (D), Sox9 (E), or Ptf1a (F). Boxed areas were enlarged and shown in the panels on the right. DAPI stained and merged images are indicated. Arrows indicate Glis3^+^Pdx1^+^ (C), Glis3^+^Ngn3^+^ (D) or Glis3^+^Sox9^+^ (E) cells; arrow heads indicate Ptf1a single positive cells (F). Triple staining of E13.5 Glis3^GFP/GFP^ pancreata with anti-GFP, anti-Nkx6.1, and Sox9 (G). Arrows indicate triple stained cells (G, inset). Dashed circles indicate tip domains; dashed lines indicate trunk domains. Schematic views (shown on the right) of the primary and secondary transition of pancreas development indicating formation of tubular structures of multipotent progenitor cells (MPC) (E12.5) and the compartmentalization and establishment of multipotent progenitors and (pre)acinar cells (A) at the tip domain and the appearance of bipotent progenitors (BP), endocrine progenitors (EP), and preductal cells (D) in the trunk domain (E13.5), respectively. DOI 10.6084/m9.figshare.3189169.

Insight into the spatiotemporal expression of Glis3 is critical for understanding the role of Glis3 during the three major periods (primary and secondary transition, and postnatal period) that delineate pancreatic development [21, 24, 36, 37]. The primary transition (embryonic day 9.5–12.5) begins with the evagination of the foregut epithelium and proliferation of pancreatic progenitors leading to the formation of the dorsal and ventral pancreatic buds. The buds subsequently develop into a network of branched tubular structures consisting of multipotent pancreatic progenitor cells (MPCs) (Pdx1^+^Ptf1a^lo^Sox9^+^Hnf1b^+^Nkx6.1^+^). Examination of Glis3-EGFP expression at these early stages of pancreas development showed that Glis3 protein was not detectable in E10.5 and E11.5 pancreas (S5 Fig) nor in the Pdx1^+^ and Sox9^+^ pancreatic progenitors at E12.5 (Fig 3A and 3B). These observations suggested that Glis3 protein is not expressed in MPCs. The start of the secondary transition (>E12.5–15.5) is accompanied by increased compartmentalization and the establishment of multipotent progenitors at the tip domain and the appearance of bipotent progenitors in the trunk domain [21, 22, 24, 36–38]. During the secondary transition, the population at the tip domain becomes heterogeneous with the MPCs steadily losing their multipotency, while the differentiation into Ptf1a^hi^ preacinar and acinar cells increases progressively. This is accompanied by a steady decrease in the expression of Sox9, Nkx6.1 and Pdx1. In contrast, the bipotent progenitors in the trunk domain lose their expression of Ptf1a, but remain Sox9^+^Nkx6.1^+^Pdx1^lo^. These cells subsequently give rise to pro-endocrine (Ngn3^hi^) and preductal cells, which then differentiate further into the different endocrine cell lineages and ductal cells, respectively. At E13.5, Glis3-EGFP fluorescence was mainly observed in the trunk domains where it overlapped largely with Pdx1^+^ (Fig 3C) and Sox9^+^ cells (Fig 3E), whereas the Ptf1a^+^ cells in the tip domains remained Glis3 negative (Fig 3F). Moreover, most Sox9^+^Nkx6.1^+^ double-positive cells, which largely represent bipotent progenitors in the trunk domains, expressed Glis3 (Fig 3G). These observations are consistent with the hypothesis that Glis3 protein is expressed in bipotent progenitors, but not in MPCs. In addition, the few Ngn3^hi^ endocrine progenitors residing in the trunk domain were all Glis3-EGFP positive indicating that Glis3 protein remains expressed in endocrine progenitors (Fig 3D).

**Fig 4:**
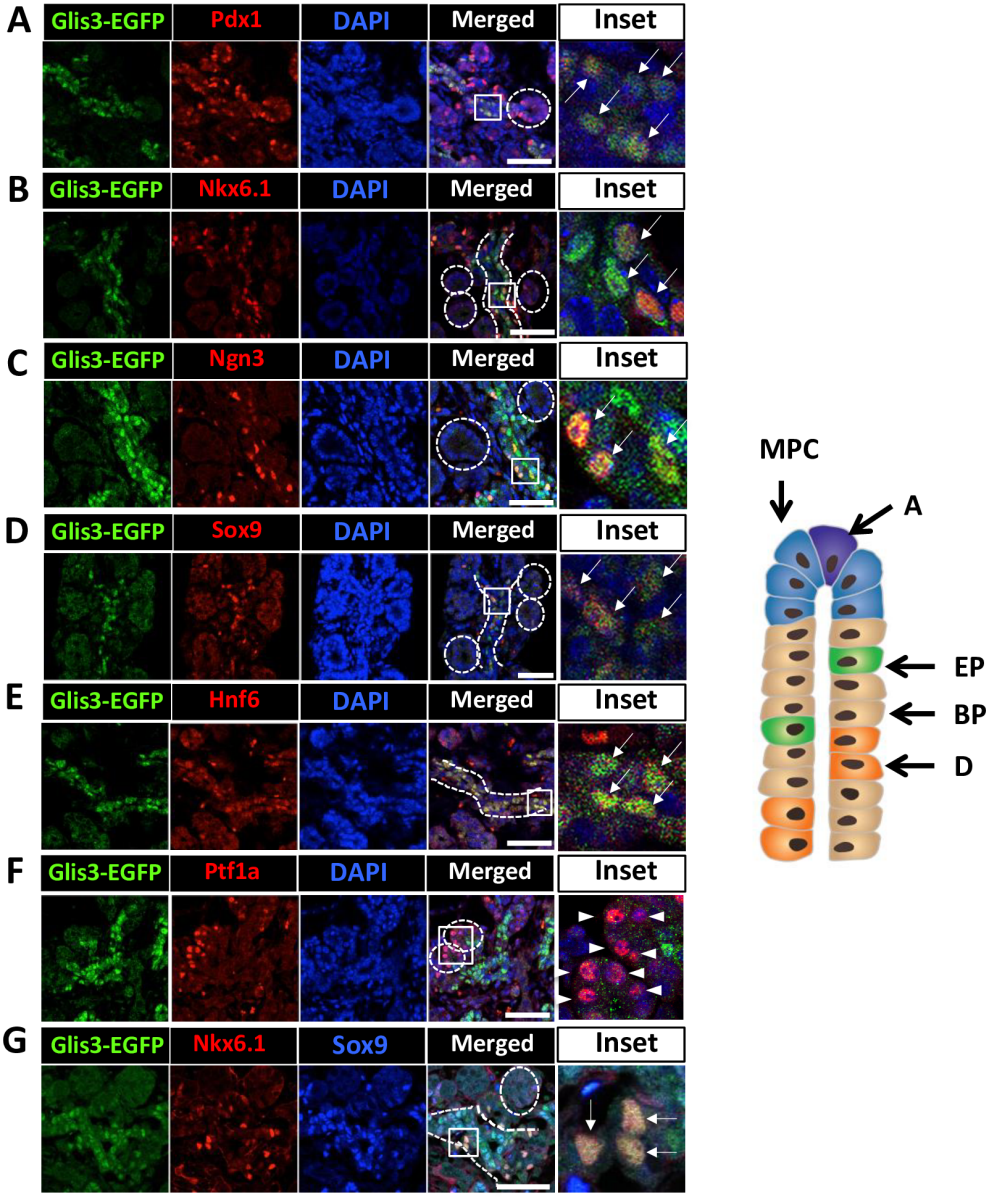
Glis3 protein expression at E15.5 of pancreas development. E15.5 Glis3^GFP/GFP^ pancreata were double-stained with antibodies against GFP (A-G) and Pdx1 (A), Nkx6.1 (B), Ngn3 (C), Sox9 (D), Hnf6 (E), or Ptf1a (M). DAPI stained and merged images are indicated. Boxed areas were enlarged and shown in the panels on the right. Arrows indicate Glis3^+^Pdx1^+^ (A), Glis3^+^Nkx6.1^+^ (B), Glis3^+^Ngn3^+^ (C), Glis3^+^Sox9^+^ (D) or Glis3^+^Hnf6^+^ (E) cells; arrow heads indicate Ptf1a single positive cells (F). Triple immunostaining of E15.5 Glis3^GFP/GFP^ pancreata with anti-GFP, anti-Nkx6.1, and Sox9 (G). Arrows indicate triple positive cells (G, inset). Dashed circles indicate tip domains; dashed lines indicate trunk domains. Schematic view of tubule structure at E15.5 of embryonic pancreatic development is indicated on the right. DOI 10.6084/m9.figshare.3189172.

Pancreatic branching and cell lineage specification continues during the later stage of the secondary transition. Bipotent progenitors are still present in the trunk and continue to give rise to (pre)ductal (Nkx6.1^-^Nng3^-^Hnf6^+^Sox9^+^) and proendocrine (Nkx6.1^+^Ngn3^+^Pdx1^+^) cells. Examination of Glis3-EGFP expression at E15.5 showed that Glis3 was not detectable in Ptf1a^hi^ cells in the tip domain (Fig 4F), but remained restricted to the trunk regions (Fig 4A–4E). In the trunk domains, Glis3 expression was observed in Hnf6^+^ (pre)ductal cells and many Sox9^+^Nkx6.1^+^ double-positive and Ngn3^hi^ endocrine progenitors (Fig 4G). More than 75 ± 4.2% of Glis3^+^ cells were Sox9^+^Nkx6.1^+^ double-positive. Nkx6.1, which is expressed in multipotent progenitors before E13, becomes restricted to bipotent, proendocrine, beta cells, and small population of α cells at E15.5 and thereafter [39]. At E15.5, Glis3 expression largely overlapped with that of Nkx6.1, but a small number of Nkx6.1 cells did not express Glis3 (Fig 4B). These observations support our conclusion that Glis3 is expressed in bipotent progenitors, endocrine progenitors, and (pre)ductal cells and is not detectable in multipotent progenitors or acinar cells.

**Fig 5:**
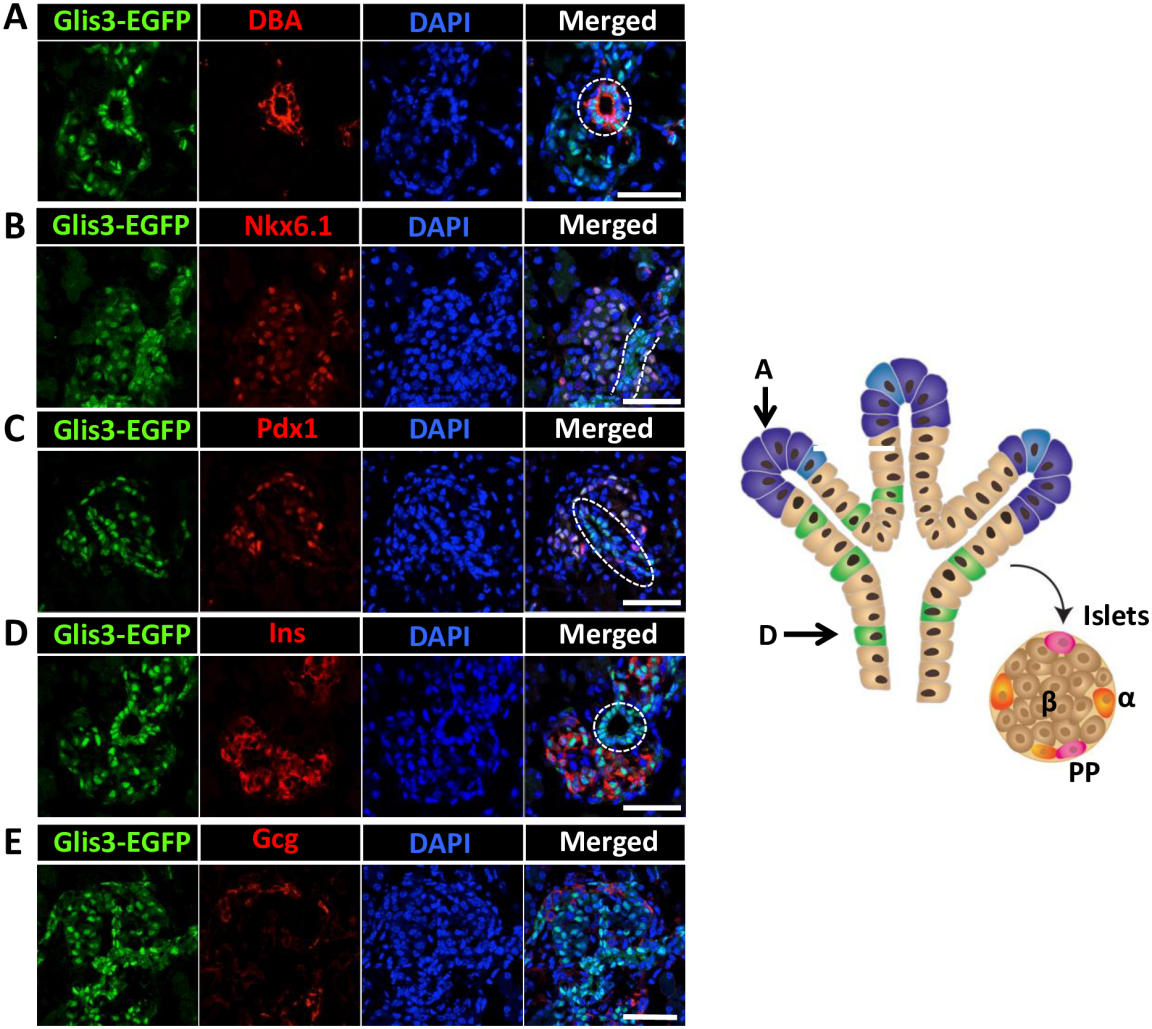
Glis3 protein expression in E17.5 pancreata. E17.5 Glis3^GFP/GFP^ pancreata were double-stained with antibodies against GFP and DBA (A), or Nkx6.1 (B), Pdx1 (C), Ins (D), or Gcg (E). DAPI and merged images are indicated. Dashed circles and dashed lines indicate pancreatic ducts. Schematic view of tubule structure and developing islets at E17.5 of embryonic pancreatic development is shown on the right: A, (pre) acinar cells; D, (pre) ductal cells; α, ß and PP cells are indicated. DOI 10.6084/m9.figshare.3189175.

Around E17.5, endocrine cells form islet structures [21, 22, 24, 36–38]. At this stage, many transcription factors exhibit a more restricted, lineage-specific pattern of expression. Glis3 continued to be expressed in DBA^+^ ductal cells, while in the majority of the cells in the islets Glis3 fluorescence greatly overlapped with the expression of Nkx6.1, Pdx1 and insulin, but was not detectable in alpha cells (Fig 5). These data are consistent with our observations in neonatal pancreas showing that Glis3 was expressed in beta cells and DBA^+^ ductal cells, but not in alpha and acinar cells (Fig 2).

### PP Cells and Ppy Are New Targets for Glis3 Regulation

**Fig 6:**
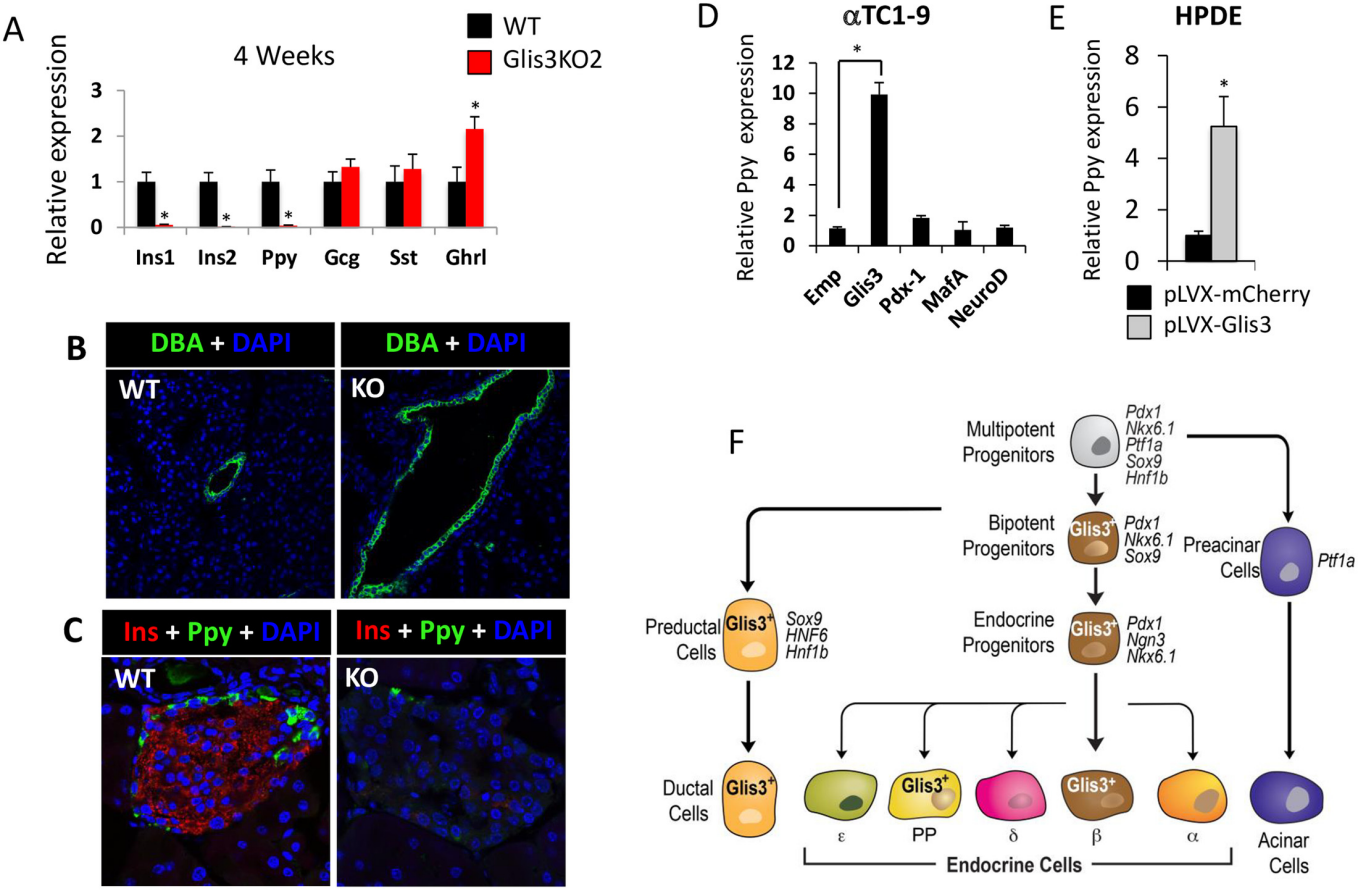
PP cells and Ppy are novel targets for Glis3 regulation. (A) Relative expression of several endocrine marker genes was analyzed by QRT-PCR in the pancreas from 4 weeks old WT and Glis3KO2 mice (n ≥ 4). (B, C) Pancreata from 4 weeks old WT and Glis3KO2 mice were examined by DAPI and FITC-DBA (B) or with antibodies against Ppy and insulin (C). (D-E) Exogenous expression of Glis3 induced Ppy mRNA expression in αTC1-9 and HPDE cells. Cells were infected with pLVX-mCherry, pLVX-mCherry-Glis3, -Pdx1, -MafA, or -NeuroD1 lentivirus and expression of Ppy mRNA was analyzed by QRT-PCR. Data present mean ± SEM. * p <0.05. (F) Schematic of the spatiotemporal pattern of expression of Glis3 during pancreas development. DOI 10.6084/m9.figshare.3189178.

The transcription factor Glis3 very likely regulates gene expression and cellular functions in cells in which it is expressed. To examine the relationship between Glis3 expression and pancreatic functions, we analyzed the effect of Glis3-deficiency on the expression of several endocrine genes in *Glis3KO2* mice. QRT-PCR analysis demonstrated that the expression of Ppy mRNA was greatly reduced in 4 weeks old Glis3KO2 pancreas (Fig 6A). Consistent with previous findings in Glis3^zf/zf^ mice [1], the expression of Ins1 and Ins2 was also greatly decreased in Glis3KO2 pancreas and loss of Glis3 expression induced development of pancreatic cysts suggesting a regulatory role in pancreatic duct morphogenesis (Fig 6B). Glucagon and somatostatin mRNA expression was not significantly altered, while the level of Ghrl mRNA was slightly enhanced (Fig 6A). The decreased expression of Ppy and insulin in Glis3KO2 islets was supported by immunohistochemistry (Fig 6C). To examine the role of Glis3 in the regulation of Ppy further, we examined whether exogenous expression of Glis3 was able to induce Ppy in αTC1-9 cells and human pancreatic ductal epithelial cells (HPDE). These cells were chosen since no PP cell line was available. Exogenous expression of Glis3 in either αTC1-9 or HPDE cells caused, respectively, a 10- and 5-fold increase in the level of Ppy mRNA expression (Fig 6D and 6E). Expression of Pdx1, MafA, or NeuroD had little effect on Ppy expression (Fig 6D). The induction of Ppy was cell type-specific since Glis3 did not induce Ppy in human kidney HEK-293 cells or the beta cell line, Min6. Together, our observations indicate that Glis3 plays a critical role controlling *Ppy* expression in PP cells in addition to its regulation of *Ins1* and *Ins2* in beta cells as well as pancreatic duct morphogenesis (Fig 6F).

## Discussion

In this study, we describe the generation of a knockin mouse expressing a Glis3-EGFP fusion protein that allowed us to analyze the spatiotemporal expression of Glis3 protein during embryonic and postnatal pancreas development. Histochemical analysis of Glis3-EGFP expression in postnatal pancreas demonstrated that Glis3 protein is expressed in a cell type-specific manner. Glis3 protein was expressed in pancreatic ducts and islets, where its expression was largely restricted to the nucleus of beta cells. We further show for the first time that Glis3 protein is expressed in PP cells where it regulates its differentiation and Ppy expression (Figs 2B and 6A). Glis3 protein was not detectable in the acini (S4 Fig). At P7 most Ins^+^ beta cells and DBA^+^ ductal cells were Glis3^+^; however, a small number of cells (about 5%) cells was Ins^+^Glis3^-^ or DBA^+^Glis3^-^. This might relate to heterogeneity within these populations at P7, a time when pancreatic ductal and beta cells expand and mature, and might involve differences in the phases of differentiation/maturation or cell cycle as has been reported for MafA [40].

Since Glis3 acts as a transcription factor, it is expected to regulate the expression of specific genes and functions in its target cells. Consistent with previous observations [1], analysis of Glis3-KO2 mice showed that loss of Glis3 function results in the formation of pancreatic cysts supporting a regulatory role for Glis3 in pancreatic duct morphogenesis. Dilation of pancreatic ducts has also been observed in mice null for other transcriptional regulators important in pancreas development, including Hnf6 and Hnf1β [41, 42]. Dilation of pancreatic ducts in Glis3-KO2 mice accompanies the development of renal cysts and both likely involve similar mechanisms that may include changes in planar cell polarity and asymmetric cell division [43]. In pancreatic beta cells, Glis3 has been reported to be a critical factor in the regulation of insulin gene transcription by binding two GlisBS in its proximal promoter [6, 9, 35]. Together, our observations indicate that Glis3 plays an important regulatory role in several pancreatic cell types in which it is expressed. Glis3 is critically involved in the regulation of *Ppy* expression in pancreatic PP cells in addition to regulating pancreatic duct morphogenesis and beta cell functions, including insulin gene transcription (Fig 6F).

We further showed that Glis3 is expressed in a spatiotemporal manner during pancreas development. The primary transition of pancreas development, which in mice starts after E8, is characterized by proliferation of MPCs and the formation and expansion of branched tubular structures at E11.5 and E12.5. Multiple transcription factors have been identified that are key regulators of early pancreas development and cell lineage determination [21, 22, 24, 36–38]. Pdx1, Hnf1β, Sox9, and Ptf1a are among the factors that play an important role in the specification and/or maintenance of MPCs [36, 42, 44, 45]. Our data show that Glis3 protein was not detectable during the primary transition at E10.5, E11.5, and E12.5, indicating that Glis3 is not expressed in MPCs (Fig 3A and 3B, and S5 Fig). This contrasts our *in situ* hybridization data showing that Glis3 mRNA was detectable in E11.5 pancreata [1]. There are several possibilities that might explain this apparent difference in Glis3 mRNA and protein expression that includes delay in Glis3 protein synthesis and accumulation due to regulation of Glis3 at a posttranscriptional level. Alternatively, Glis3 protein might be unstable in MPCs or may be in a protein complex that is not accessible by the anti-EGFP antibody.

At E13.5, at the beginning of the secondary transition, cells increasingly compartmentalize and MPCs become restricted to the tip domain where they progressively lose their multipotency and differentiate into preacinar and subsequently acinar cells [21, 22, 24, 36–38]. This is accompanied by a steady increase in Ptf1a expression and decrease in Pdx1 and Nkx6.1. Mutual repression between Nkx6.1 and Ptf1a has been reported to be one key factor in the acinar versus bipotent cell lineage determination [45]. At E13.5 and E15.5, Glis3-EGFP was not detectable at the tip domain consistent with our conclusion that Glis3 is not expressed in MCPs and (pre)acinar cells (Figs 3F and 4F). Early in the secondary transition cells in the trunk domain give rise to endocrine-duct bipotent progenitors, which is accompanied by a down-regulation of Ptf1a expression and an increase in Sox9 [21, 22, 24, 36, 38]. At E13.5, Glis3 is detectable in most cells within the trunk domain suggesting that it is expressed in bipotent progenitors (Fig 3C–3E). This hypothesis is supported by data showing that at E13.5 most Sox9^+^Nkx6.1^+^ cells are Glis3^+^ (Fig 3G). The bipotent progenitors subsequently differentiate into Ngn3^+^ proendocrine progenitors and DBA^+^ (pre)ductal cells, both of which express Glis3^+^ (Figs 4C and 5A). This expression pattern is consistent with a role for Glis3 in promoting the generation of Ngn3^+^Glis3^+^ endocrine progenitors from Glis3^+^ bipotent progenitors [5, 31] and in the transcriptional regulation of Ngn3 expression. Whether Glis3 regulates a specific stage of endocrine progenitor cell differentiation or the proliferation of these cells has yet to be determined.

In the developing islets at E17.5, Pdx1 and Nkx6.1 become mainly restricted to Ins^+^ beta cells where their expression greatly overlaps with that of Glis3 as was observed in neonatal pancreas (Fig 5A–5D). Thus, during the differentiation of the Ngn3^+^Glis3^+^ endocrine progenitors into several endocrine cell lineages, Glis3 expression is maintained in pancreatic beta and PP cells, but is repressed in alpha, delta and epsilon cells (Figs 2A–2E and 5E). This raises several interesting questions: Does Glis3 have a role in lineage determination and is repression of Glis3 required for the differentiation of endocrine progenitors into alpha and delta cells? Repression of Glis3 in α cells might be mediated by α cell-specific transcription factors, such as Arx and/or Foxa1/2. Preliminary data showing that exogenous expression of Arx or Foxa1 reduced Glis3 expression in beta cells support this hypothesis (Kang, H.S. and Jetten, A.M., unpublished observations). Inversely, constitutive expression of Glis3 might repress the differentiation of endocrine progenitors into alpha and delta cells. This possibility is currently under investigation. Recently it has been shown that PP cells are originated mainly from Ngn3^+^ endocrine progenitor cells as well as Ghrl^+^ cells [46]. The expression of Glis3-EGFP was barely detectable in Ghrl^+^ cells at P7 pancreas (Fig 2E), and the expression of Ghrl was not much different between WT and Glis3-KO2 at P7 pancreas (S6 Fig). This result suggests that Glis3 may not play a major role in Ghrl cell lineage determination and that small population of PP cells in Glis3-KO2 mice could be originated from formerly Ghrl produced cells.

Together, our study demonstrates that Glis3 exhibits a temporal and cell type-specific pattern of expression during embryonic and neonatal pancreas development. These findings are consistent with a regulatory function for Glis3 in bipotent and endocrine progenitors, including the generation of endocrine progenitors and the regulation of Ngn3 expression during early pancreas development. As well as in the control of pancreatic beta, PP, and ductal cell functions, including the regulation of insulin and Ppy expression, and pancreatic duct morphogenesis (Fig 6E). These observations will help us to better understand the role of Glis3 in the pancreas and in the development of diabetes.

## Supporting Information

S1 Fig Comparison of Glis3 and Glis3-EGFP mRNA expression in WT, Glis3^GFP/GFP^, Glis3^+/GFP^ mice. RNA was isolated from WT, Glis3^GFP/GFP^, Glis3^+/GFP^ mouse kidney and examined by Northern blot analysis with [^32^P]-labeled Glis3 or EGFP probes. 28S and 18S rRNA were used as loading controls. The radiolabeled EGFP probe hybridized only to a 9 kb RNA in kidney samples from Glis3^GFP/GFP^ and Glis3^+/GFP^ mice. The [^32^P]-labeled Glis3 and EGFP probes hybridized to a single Glis3-EGFP transcript with the expected size of about 9 kb, slightly larger than of the WT Glis3 transcript. The radiolabeled EGFP probe hybridized only to a 9 kb RNA in kidney samples from Glis3^GFP/GFP^ and Glis3^+/GFP^ mice. DOI 10.6084/m9.figshare.3189181.
(TIF)

S2 Fig The majority of the cells in pancreatic islets expressed Glis3. Sections of PND7 pancreata from Glis3^GFP/GFP^ mice were stained with DAPI and anti-GFP and anti-E-cadherin (Ecad) antibodies. Expression of Glis3 is largely restricted to the nucleus. DOI 10.6084/m9.figshare.3189184.
(TIF)

S3 Fig The majority of insulin positive cells expressed Glis3. Sections of PND7 pancreata from Glis3^GFP/GFP^ mice were stained with DAPI and anti-GFP and anti-insulin antibodies. * indicates Ins^+^Glis3^-^ cells, arrow indicates Ins^-^Glis3^+^, and dashed circles indicate ductal cells. DOI 10.6084/m9.figshare.3189187.
(TIF)

S4 Fig Pancreatic acini do not stain for Glis3-EGFP. Sections of P7 pancreas Glis3^GFP/GFP^ embryos were stained with anti-GFP and anti-amylase antibodies. DOI 10.6084/m9.figshare.3189190.
(TIF)

S5 Fig Glis3 protein was not detectable in E10.5 or E11.5 pancreata. Sections of E10.5 and E11.5 Glis3^GFP/GFP^ embryos were stained with anti-GFP and anti-Pdx1 antibodies. Glis3 was not detectable in Pdx1^+^ cells. DOI 10.6084/m9.figshare.3189193.
(TIF)

S6 Fig Staining for ghrelin (Ghrl) was not significantly different between P7 pancreas of WT and Glis3-KO2 mice. Sections of P7 pancreata from WT and Glis3-KO2 mice were stained with DAPI, anti-Ghrl and anti-Pdx1 antibodies. DOI 10.6084/m9.figshare.3189196.
(TIF)

S1 Table List of QRT-PCR primers. (XLSX)

## Abbreviations

DBA: lectin Dolichos biflorus agglutinin
EGFP: enhanced green fluorescence protein
Gcg: Glucagon
Ins: Insulin
MPC: pancreatic progenitor cells
NDH: neonatal diabetes and hypothyroidism
Ppy: pancreatic polypeptide
Sst: Somatostatin
