# Supplementary figures and images for: The Spatiotemporal Pattern of Glis3 Expression Indicates a Regulatory Function in Bipotent and Endocrine Progenitors during Early Pancreatic Development and in Beta, PP and Ductal Cells
Source: PLoS One. 2016 Jun 7;11(6):e0157138. doi: 10.1371/journal.pone.0157138 (PMC4896454; doi:10.1371/journal.pone.0157138)

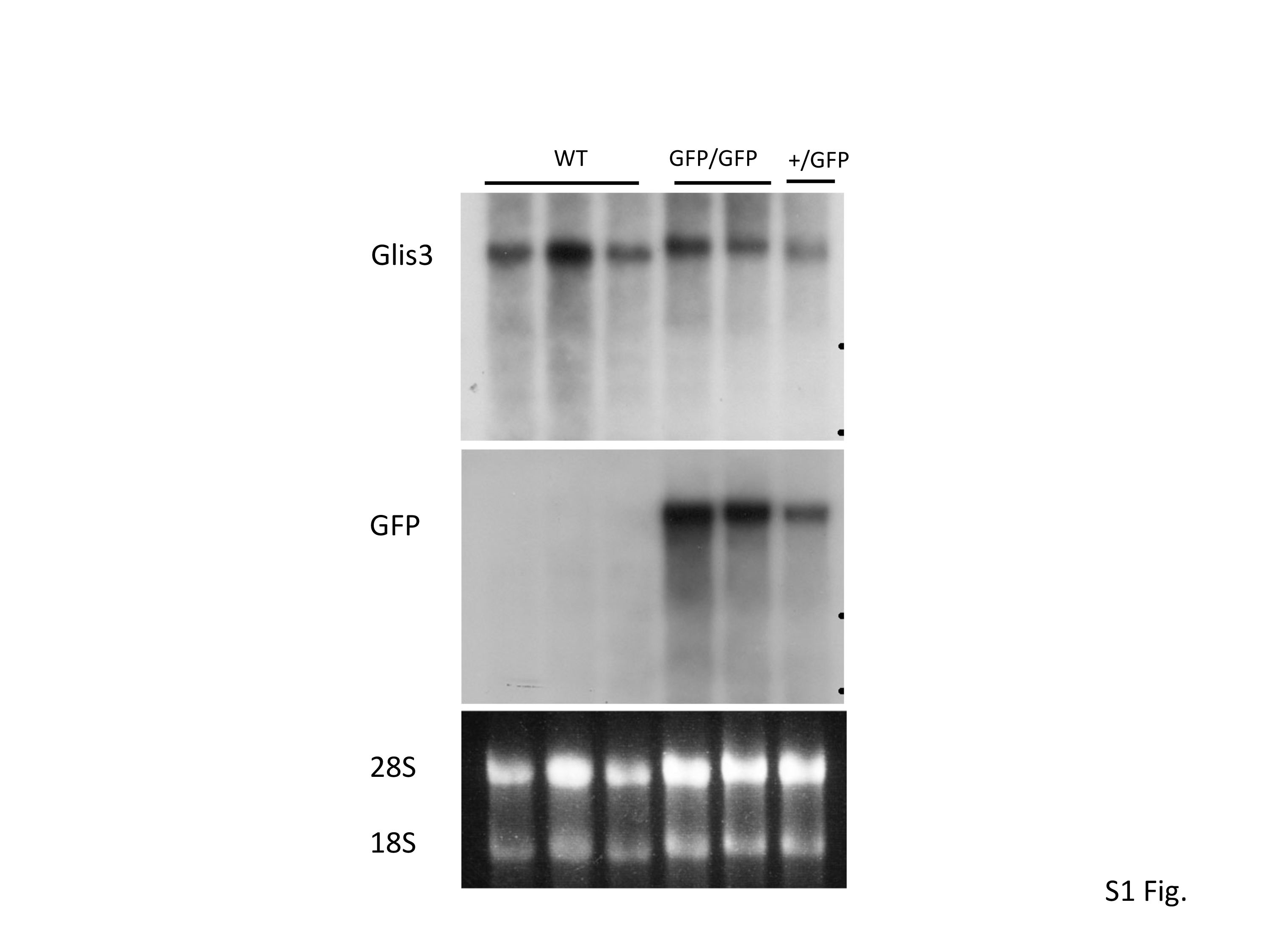

Supplement: S1 Fig — RNA was isolated from WT, Glis3GFP/GFP, Glis3+/GFP mouse kidney and examined by Northern blot analysis with [32P]-labeled Glis3 or EGFP probes. 28S and 18S rRNA were used as loading controls. The radiolabeled EGFP probe hybridized only to a 9 kb RNA in kidney samples from Glis3GFP/GFP and Glis3+/GFP mice. The [32P]-labeled Glis3 and EGFP probes hybridized to a single Glis3-EGFP transcript with the expected size of about 9 kb, slightly larger than of the WT Glis3 transcript. The radiolabeled EGFP probe hybridized only to a 9 kb RNA in kidney samples from Glis3GFP/GFP and Glis3+/GFP mice. DOI 10.6084/m9.figshare.3189181. (TIF) [file pone.0157138.s001.tif]

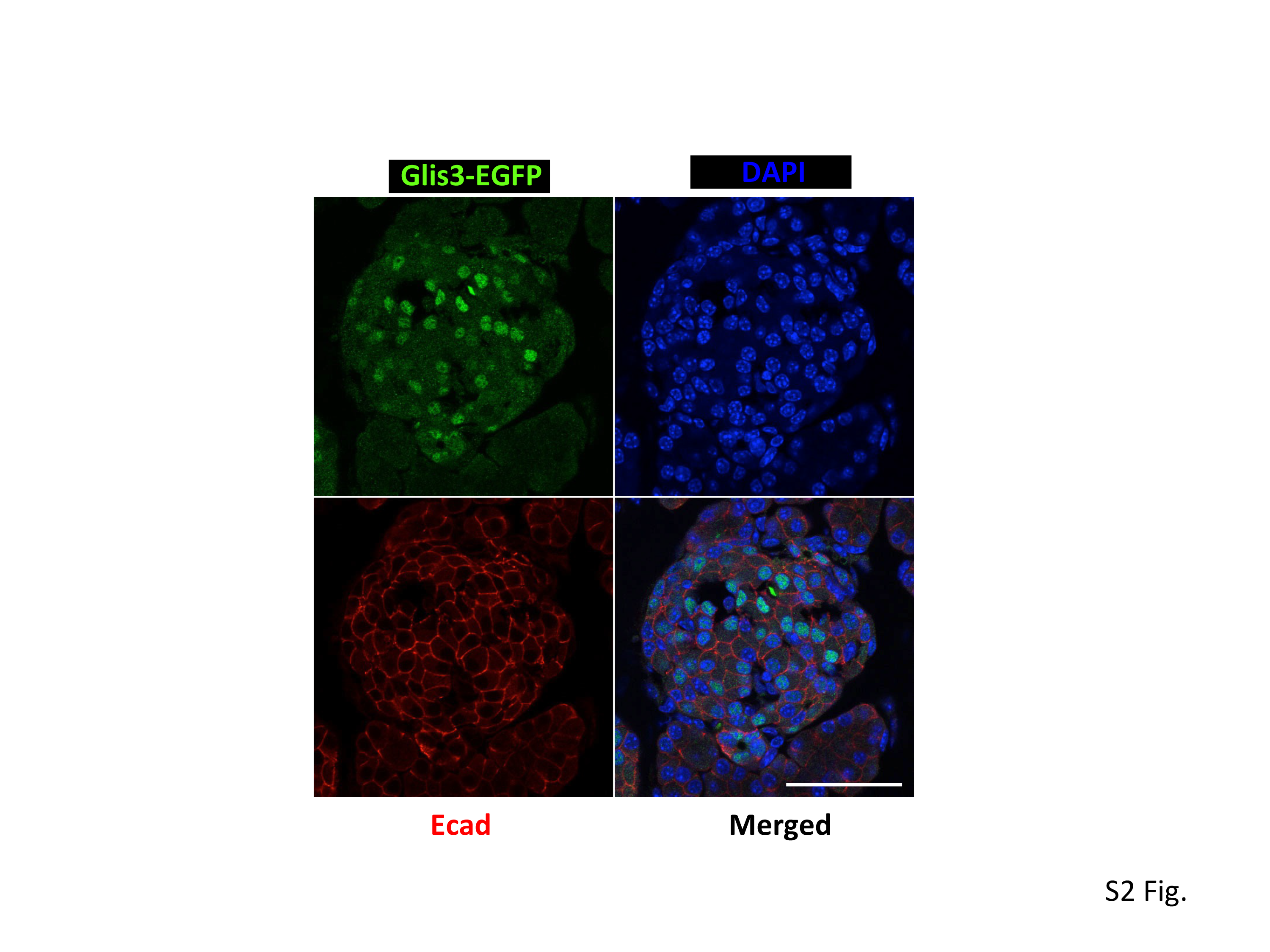

Supplement: S2 Fig — Sections of PND7 pancreata from Glis3GFP/GFP mice were stained with DAPI and anti-GFP and anti-E-cadherin (Ecad) antibodies. Expression of Glis3 is largely restricted to the nucleus. DOI 10.6084/m9.figshare.3189184. (TIF) [file pone.0157138.s002.tif]

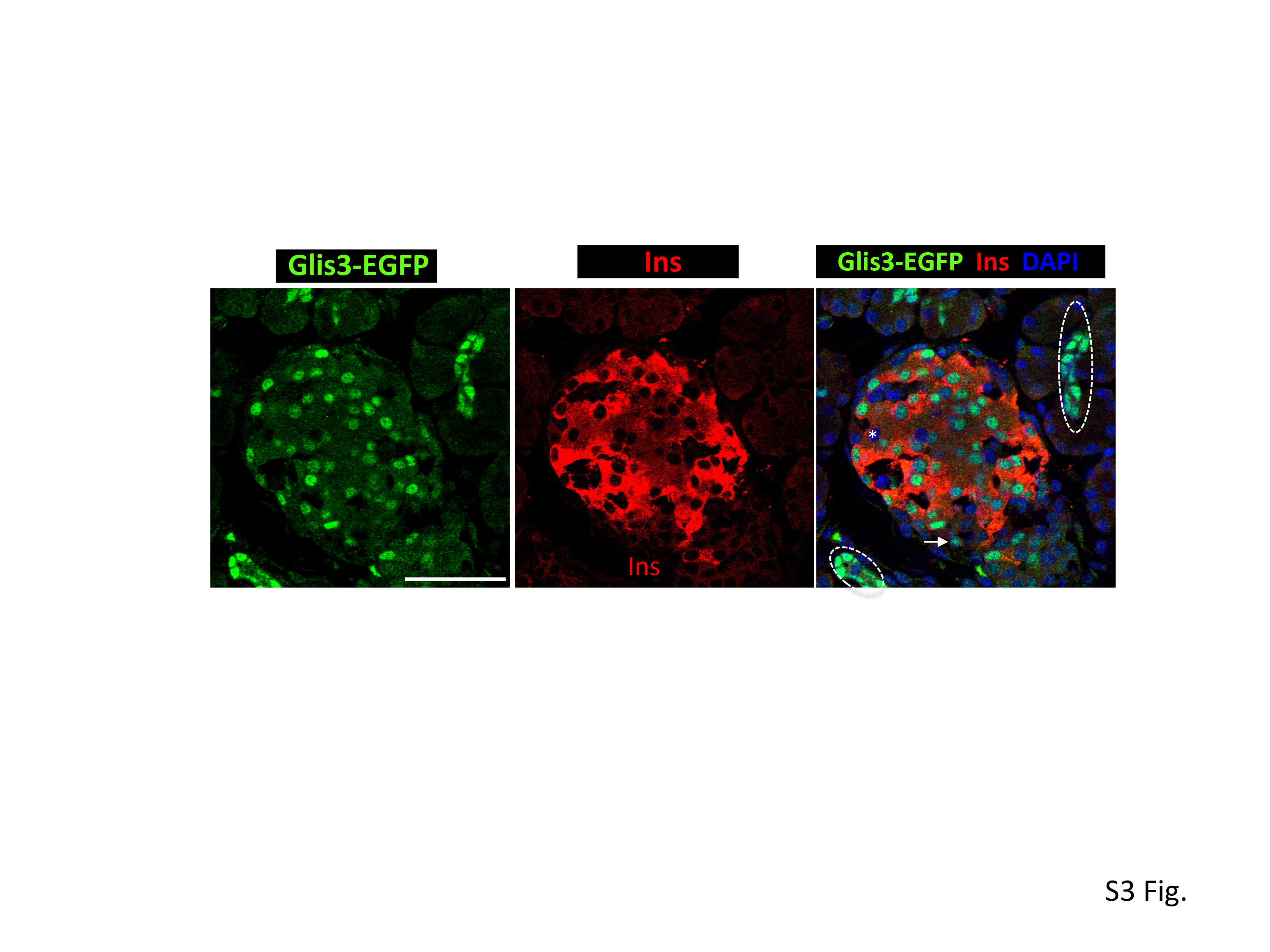

Supplement: S3 Fig — Sections of PND7 pancreata from Glis3GFP/GFP mice were stained with DAPI and anti-GFP and anti-insulin antibodies. * indicates Ins+Glis3- cells, arrow indicates Ins-Glis3+, and dashed circles indicate ductal cells. DOI 10.6084/m9.figshare.3189187. (TIF) [file pone.0157138.s003.tif]

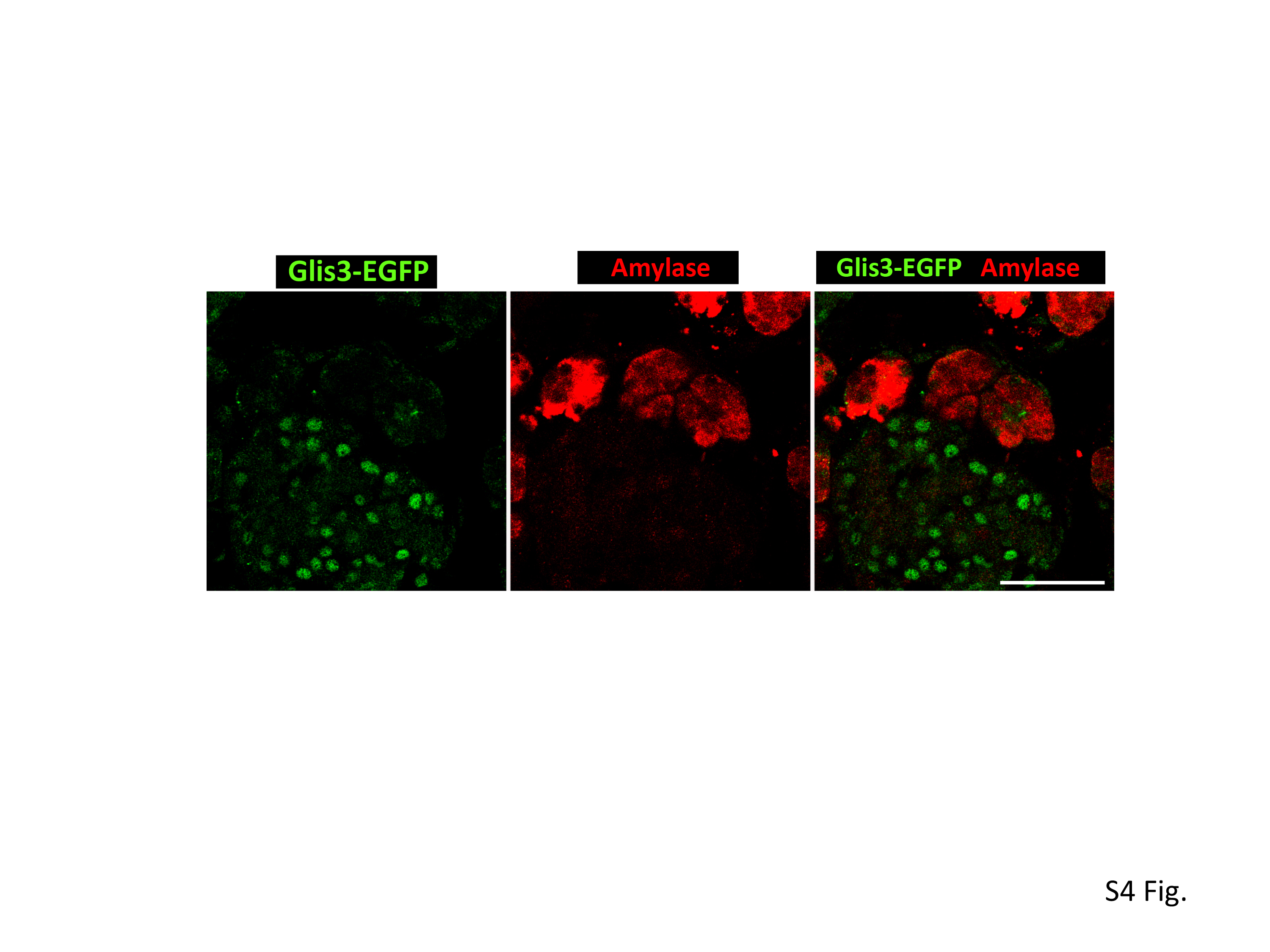

Supplement: S4 Fig — Sections of P7 pancreas Glis3GFP/GFP embryos were stained with anti-GFP and anti-amylase antibodies. DOI 10.6084/m9.figshare.3189190. (TIF) [file pone.0157138.s004.tif]

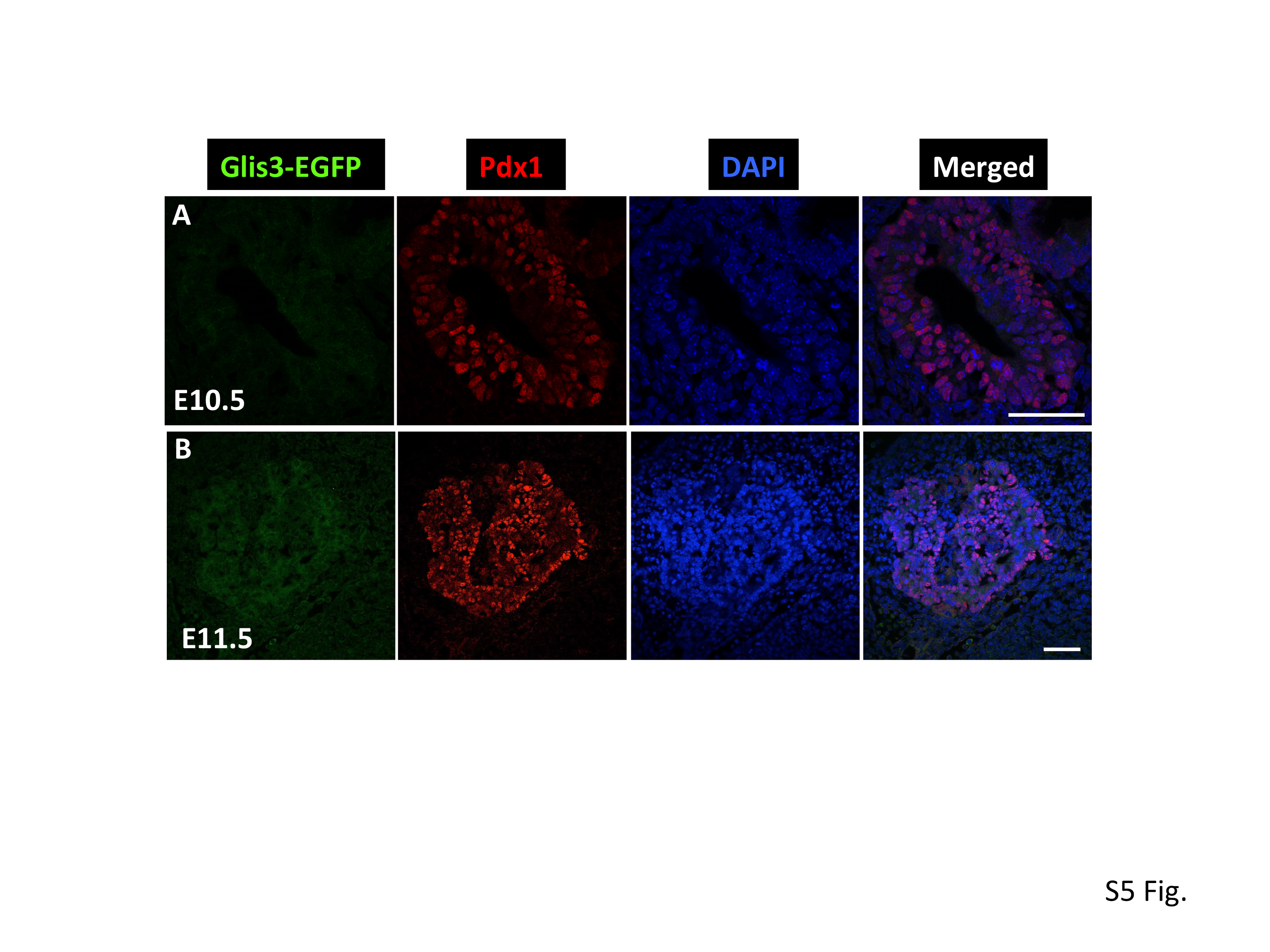

Supplement: S5 Fig — Sections of E10.5 and E11.5 Glis3GFP/GFP embryos were stained with anti-GFP and anti-Pdx1 antibodies. Glis3 was not detectable in Pdx1+ cells. DOI 10.6084/m9.figshare.3189193. (TIF) [file pone.0157138.s005.tif]

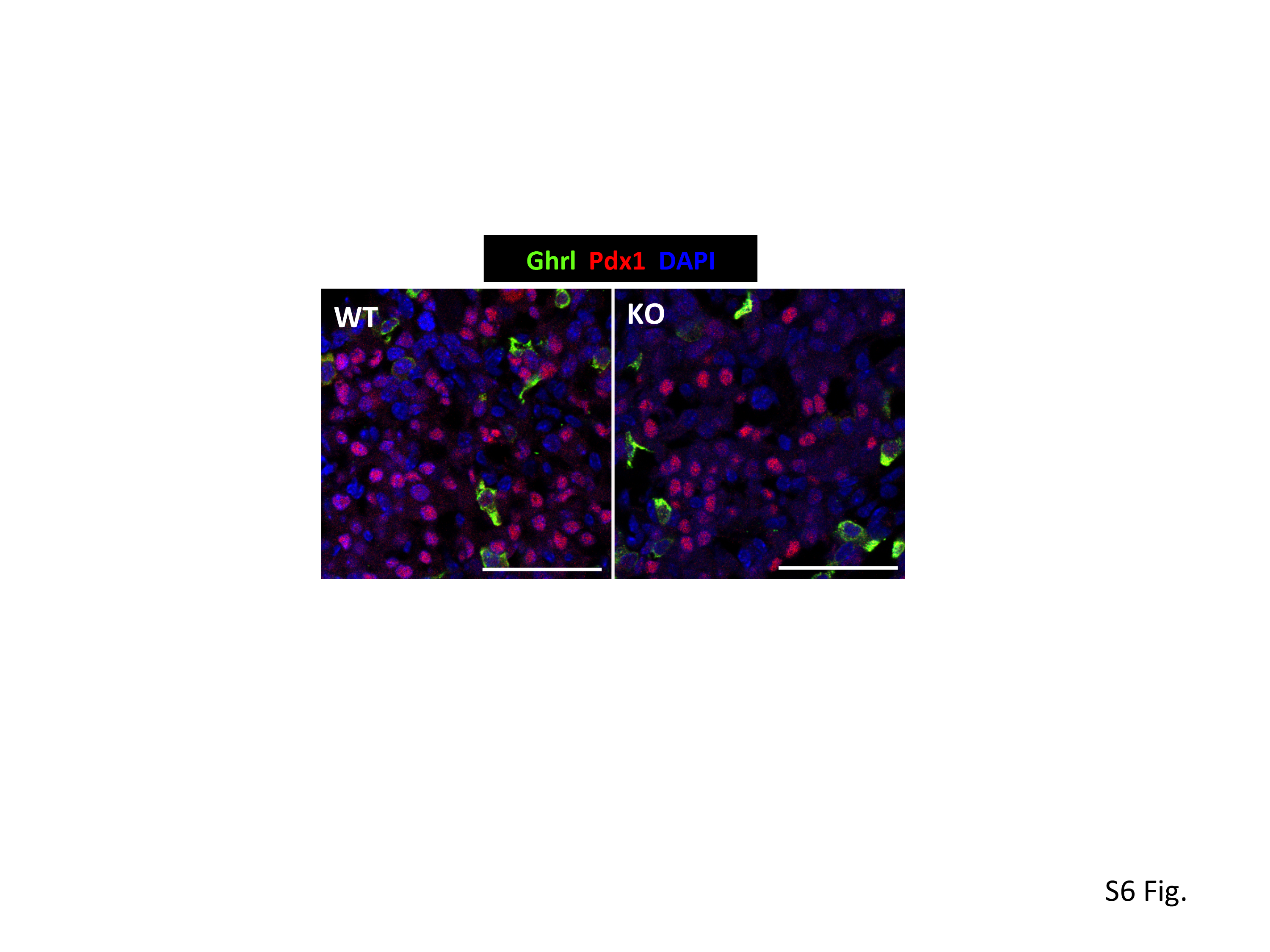

Supplement: S6 Fig — Sections of P7 pancreata from WT and Glis3-KO2 mice were stained with DAPI, anti-Ghrl and anti-Pdx1 antibodies. DOI 10.6084/m9.figshare.3189196. (TIF) [file pone.0157138.s006.tif]
